# Complete mitochondrial genome of the surf smelt *Hypomesus japonicus* (Osmeriformes, Osmeridae)

**DOI:** 10.1080/23802359.2018.1511852

**Published:** 2018-09-10

**Authors:** Evgeniy S. Balakirev, Nikolai S. Romanov, Francisco J. Ayala

**Affiliations:** a Department of Ecology and Evolutionary Biology, University of California , Irvine , CA , USA ;; b National Scientific Center of Marine Biology, Far Eastern Branch, Russian Academy of Sciences , Vladivostok , Russia ;; c School of Natural Sciences, Far Eastern Federal University , Vladivostok , Russia

**Keywords:** Surf smelt *Hypomesus japonicus*, Japanese smelt *Hypomesus nipponensis*, pond smelt *Hypomesus olidus*, Arctic rainbow smelt *Osmerus dentex*, European smelt *O. eperlanus*, Atlantic rainbow smelt *O. mordax*, Osmeridae, *Mallotus*, *Hypomesus*, *Osmerus*, mitochondrial genome

## Abstract

The complete mitochondrial genome was sequenced in two individuals of the surf smelt *Hypomesus japonicus*. The genome sequences are 16,762 and 16,771 bp in size, and the gene arrangement, composition, and size are very similar to the other smelt mitochondrial genomes published previously. The difference between two *H. japonicus* genomes studied is 0.37%, which is noticeably higher in comparison with other osmerid fishes. The level of sequence divergence between *H. japonicus* and related osmerids belonging to genera *Hypomeus*, *Osmerus*, and *Mallotus* varies within a very narrow range (12.31–13.72%) indicating poor phylogenetic resolution of this complex fish group.

The surf smelt *Hypomesus japonicus* (Brevoort) is a coastal osmerid fish inhabiting the northwestern Pacific Ocean including the Bering Sea, the Sea of Okhotsk, and the Sea of Japan (Pietsch et al. 2001). The taxonomy of the genus *Hypomesus* is highly controversial (Shedko 2001 and references therein). Despite intensive investigations based on morphological characteristics (e.g. Berg 1949; Klyukanov 1970; review in Shedko 2001), the species composition and taxonomy of the genus *Hypomesus* are not clear. The traditional taxonomy based on morphological characteristics is not congruent with the genetic data, which could be explained by the homoplasious morphological characters used in previous studies (Ilves and Taylor 2009).

To increase the power of the molecular taxonomy analysis of this complex fish group, we have sequenced two complete mitochondrial (mt) genomes of *H. japonicus* (GenBank accession numbers MH636616 and MH636617) from the Amur Bay of the Sea of Japan (43°13′17,256″ N; 131°55′37,113″ E; 07.01.2014). The primers were designed with the program mitoPrimer_V1 (Yang et al. 2011). The fish specimens are stored at the museum of the National Scientific Center of Marine Biology, Vladivostok, Russia (www.museumimb.ru) under accession numbers MIMB35008 and MIMB35009.

The *H. japonicus* mt genome sequences are 16,762 and 16,771 bp in size and the gene arrangement, composition, and size are very similar to the smelt fish genomes published previously. We detected 62 single nucleotide and four length differences between the haplotypes 390Hj1 and 392Hj3; total sequence divergence (*D*_xy_) was 0.0037 ± 0.0005. The difference between the two *H. japonicus* mt genomes studied is relatively high in comparison with close species, the European smelt *O. eperlanus* (*D*_xy_ = 0.0005 ± 0.0001) and Arctic rainbow smelt *Osmerus dentex* (*D*_xy_ = 0.0025 ± 0.0004) (Balakirev et al. 2018a, 2018b).

The comparison of the mt genomes now obtained with other complete mt genomes of related groups available in GenBank including genera *Hypomesus*, *Osmerus*, *Mallotus*, *Hemisalanx*, and *Salanx* reveals a close affinity of *H. japonicus* to other *Hypomesus* species (Figure 1). The difference between *H. japonicus* and the cluster of *H. nipponensis + H. olidus* is high enough (*D*_xy_ = 0.1269 ± 0.0019) to consider *H. japonicus* as a separate biological species. However, the difference (*D*_xy_) between *H. nipponensis* and *H. olidus* is 0.0113 ± 0.0007, which is 8.6 times lower than the average level of divergence (*D*_xy_ = 0.0971 ± 0.0014) between five available smelts genomes (genera *Osmerus*, *Mallotus*, and *Hypomesus*) excluding *H. nipponensis* and *H. olidus*, and which could be explained by erroneous species identification (Balakirev et al. 2017) or interspecific replacement of mtDNA (Bernatchez et al. 1995). The level of sequence divergence between *H. japonicus* and other osmerid fishes, *H. nipponensis*, *H. olidus*, *Osmerus dentex*, *O. mordax*, *O. eperlanus*, and *Mallotus villosus* varies in a very narrow range (12.34–13.09%) indicating poor phylogenetic resolution of this group based on the mt sequences; a problem previously discussed (Ilves and Taylor 2009; Skurikhina et al. 2013).

**Figure 1.**
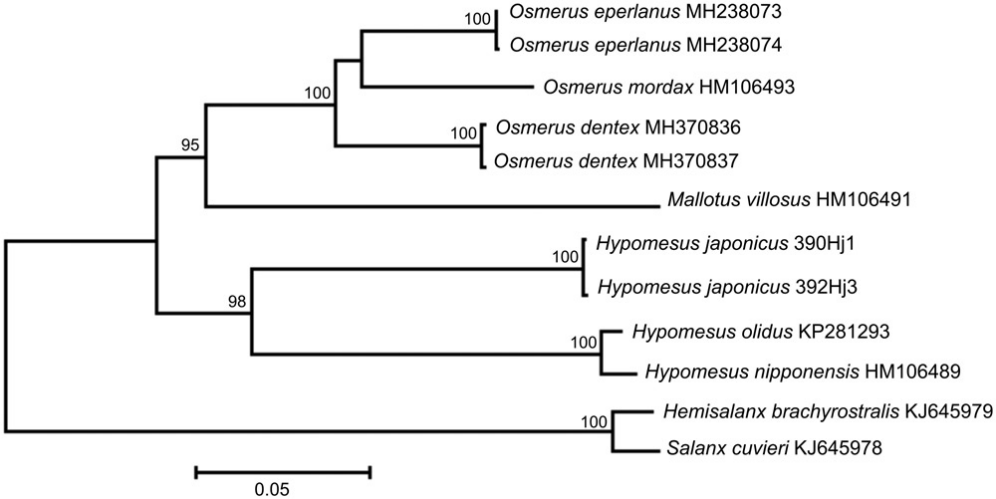
Maximum likelihood tree for the surf smelt *Hypomesus japonicus* specimens 390Hj1 and 392Hj3, and GenBank representatives of the order Osmeriformes. The tree is based on the General Time Reversible*+* gamma*+* invariant sites (GTR + G+I) model of nucleotide substitution. The numbers at the nodes are bootstrap percent probability values based on 1000 replications (values below 75% are omitted).
